# Case Report: Phantom limb pain relief after cognitive multisensory rehabilitation

**DOI:** 10.3389/fpain.2024.1374141

**Published:** 2024-04-25

**Authors:** Marina Zernitz, Carla Rizzello, Marco Rigoni, Ann Van de Winckel

**Affiliations:** ^1^ Centro Studi di Riabilitazione Neurocognitiva, Villa Miari, Vicenza, Italy; ^2^ Division of Physical Therapy and Rehabilitation Science, Department of Family Medicine and Community Health, Medical School, University of Minnesota Twin Cities, Minneapolis, MN, United States

**Keywords:** phantom limb pain, mental body representations, multisensory motor imagery, neurological rehabilitation, cognitive multisensory rehabilitation, amputation, lower limb, adults

## Abstract

**Introduction:**

Relieving phantom limb pain (PLP) after amputation in patients resistant to conventional therapy remains a challenge. While the causes for PLP are unclear, one model suggests that maladaptive plasticity related to cortical remapping following amputation leads to altered mental body representations (MBR) and contributes to PLP. Cognitive Multisensory Rehabilitation (CMR) has led to reduced pain in other neurologic conditions by restoring MBR. This is the first study using CMR to relieve PLP.

**Methods:**

A 26-year-old woman experienced excruciating PLP after amputation of the third proximal part of the leg, performed after several unsuccessful treatments (i.e., epidural stimulator, surgeries, analgesics) for debilitating neuropathic pain in the left foot for six years with foot deformities resulting from herniated discs. The PLP was resistant to pain medication and mirror therapy. PLP rendered donning a prosthesis impossible. The patient received 35 CMR sessions (2×/day during weekdays, October–December 2012). CMR provides multisensory discrimination exercises on the healthy side and multisensory motor imagery exercises of present and past actions in both limbs to restore MBR and reduce PLP.

**Results:**

After CMR, PLP reduced from 6.5–9.5/10 to 0/10 for neuropathic pain with only 4–5.5/10 for muscular pain after exercising on the Numeric Pain Rating Scale. McGill Pain Questionnaire scores reduced from 39/78 to 5/78, and Identity (ID)-Pain scores reduced from 5/5 to 0/5. Her pain medication was reduced by at least 50% after discharge. At 10-month follow-up (9/2013), she no longer took Methadone or Fentanyl. After discharge, receiving CMR as outpatient, she learned to walk with a prosthesis, and gradually did not need crutches anymore to walk independently indoors and outdoors (9/2013). At present (3/2024), she no longer takes pain medication and walks independently with the prosthesis without assistive devices. PLP is under control. She addresses flare-ups with CMR exercises on her own, using multisensory motor imagery, bringing the pain down within 10–15 min.

**Conclusion:**

The case study seems to support the hypothesis that CMR restores MBR which may lead to long-term (12-year) PLP reduction. MBR restoration may be linked to restoring accurate multisensory motor imagery of the remaining and amputated limb regarding present and past actions.

## 1 Introduction

Phantom limb pain (PLP) after amputation is defined as neuropathic pain in the missing body part (1, 2). Neuropathic pain is described as burning, stabbing, shooting, throbbing, and “pins and needles” (3). Up to 80% of adults with an amputation have phantom limb pain (PLP) (3–5), usually reported within the first week after amputation (3). Secondary effects include depression, impairments in daily life activities, and decreased quality of life (3, 4, 6).

Current treatments for PLP reduction include pain medication, peripheral nerve stimulation, repetitive transcranial magnetic stimulation, transcranial direct current stimulation, graded motor imagery, mirror therapy, phantom motor execution, virtual and augmented reality, eye movement desensitization and reprocessing, as well as surgical methods such as targeted muscle reinnervation and regenerative peripheral nerve interfaces (3, 7–21). However, reviews and clinical trials on these therapies demonstrate mixed results, with heterogeneity among patients and little evidence to support efficacy due to small sample studies, poor methodological quality, and lack of long-term follow-up periods (3, 7–20, 22, 23).

The mechanisms contributing to this type of pain are not fully understood, but one of the most common theoretical models is the cortical remapping theory driven by a deafferentation-related disinhibition, where PLP is the result of maladaptive plasticity with notable changes in the sensory and motor cortex related to the amputated limb, as well as preserved representation of the amputated limb (23–26). Additionally, cognitive and psychological factors are involved in the network-level organization maintaining the phantom limb pain (24).

Finally, alterations in mental body representations (MBR) are worth considering within the development and maintenance of PLP. It is well established that a dynamic flexible MBR is constructed in the brain based on the integration of multisensory information (27, 28), and that the secondary sensory somatosensory cortex (i.e., parietal operculum), the superior parietal lobe, and the insula are involved in MBR (24, 29, 30). Disturbed MBR processing due to impaired multisensory integration following amputation may contribute to persistent chronic pain (31). Moreover, learning-related and memory-related plastic changes of the central nervous system with concomitant maladaptive changes in body perception can be seen in adults with chronic pain (31).

Cognitive multisensory rehabilitation (CMR) is a rehabilitation approach that focuses on restoring MBR (29, 30, 32), thereby offering a unique approach to alleviating PLP. CMR distinguishes itself from other treatments such as regular sensory discrimination tasks, motor imagery, mirror therapy, and virtual or augmented reality because the therapist provides the patient with specific questions to restore their MBR. Those questions invite the patient to access and restore the multisensory imagery between the remaining limb and imagery of amputated limb, or make comparisons between present and past actions, comparisons between current tactile sensations of different textures and past memories in the remaining limb, etc. This is in contrast to the other approaches where comparisons in the mirror, virtual, or augmented reality are meant to trick the brain into believing the amputated limb is still there, or in the case of regular sensory discrimination tasks where the learning happens through trial and error. During CMR, the therapist helps the patient remember and reconstruct this multisensory imagery of the remaining and amputated limb in present and past actions. In other words, rather than unlearning a pain response using specific operant-based extinction training (31), or using *perceived* reality in the case of mirror therapy, virtual or augmented therapy (23), CMR uses *actual* past memories and *actual* present moment imagery and sensations in the amputated and other limb to restore the MBR, and through the comparison questions, the therapist helps the patient access and restore those memories and imageries.

Research on the effects of CMR demonstrated improved sensorimotor and visual recovery after cortical blindness (33), sensorimotor recovery after stroke (29, 34–37), and shoulder impingement syndrome alleviation (38). Our prior research in adults with spinal cord injury (SCI)-related neuropathic pain has demonstrated that 6 weeks of CMR (3×/week, 45 min/session) led to significant pain reduction and improved sensorimotor function, maintained at 1-year follow-up (30, 32). These improvements were seen alongside stronger resting-state parietal operculum connectivity and increased brain activation in areas related to pain, MBR, and sensory function during a toe stimulation fMRI task (30, 32). Therefore, based on the fact that adults with SCI-neuropathic pain and adults with PLP are thought to share some similar neurophysiological mechanisms in terms of cortical reorganization and the association between brain reorganization and pain intensity (39), we hypothesize similar effects can be obtained with CMR in adults with PLP. This case report presents the results of CMR in a patient who suffered from severe PLP after experiencing failed results with medications, epidural stimulation, and mirror therapy. PLP hindered her from donning a prosthesis and walking.

## 2 Case description

The patient was a 26-year-old non-smoking female student with a normal BMI who experienced six years of severe neuropathic pain in her left foot with foot deformities resulting from herniated discs (L4–L5, L5–S1). She received an epidural stimulator, underwent several surgeries, and took high doses of analgesics, but all were unsuccessful in reducing her pain. Ultimately, a below-knee amputation was performed on June 28, 2012, which led to excruciating PLP. Mirror therapy exacerbated the pain. Touching the stump was so excruciatingly painful that donning a prosthesis was impossible. Three months and two days after the amputation, she was admitted to the rehabilitation center “Study Center of Neurocognitive Rehabilitation” (Villa Miari, Italy) to receive CMR. At that time, she was dependent on assistance for daily living. Her social life and education were severely affected. PLP hindered her sleep. Details of the timeline of events are described in Table 1. The study followed the principles of the Declaration of Helsinki (40). Case studies are exempt from ethical review as per regulations according to the Ethical Committee of the South-East Venice Territorial Area (Comitato Etico Territoriale Area Sud-Ovest Veneto). The person signed informed consent and HIPAA requirements were met. The patient also signed consent for the pictures presented in the Supplementary Material.

**Table 1:** Timeline of events of symptoms, and treatment.

Timeline of events	Interventions and results
December 2006	Herniated disc (L4–L5, L5–S1) during a lifting maneuver. RESULT: Pain and weakness when performing plantar flexion with the left foot. Increased numbness in the big toe and 2nd toe as well as the rear and medial side of the left foot.
March 2007	These impairments progressed towards an initial talipes equinovarus deformity of the left foot.
April 2007	The ankle was unlocked under anesthesia. Two different casts were applied. RESULT: Removal of casts because the patient did not tolerate them.
End of April 2007	Diagnosis of reflex sympathetic algoneurodystrophy, a dystonic posture of the left foot with contracture of the posterior tibialis muscle.
May 2007	Placement of analgesic epidural catheter. RESULT: Pain decreased; deformity of the foot worsened; and oedema increased. The patient was unable to bear weight on the foot and used crutches to walk.
March 2009	The patient is fitted with an epidural stimulator.
April 2009	Achilles tenotomy was performed to correct the settled talipes equinovarus deformity of the left foot. Ilizarov fixators were applied for gradual correction over one year.
April 2010	RESULT: The foot was still in an equinus position and slightly internally rotated.
April 2011	The patient underwent triple arthrodesis of the ankle with screws, as well as a tenotomy of the toe flexor and plantar fascia. The toes were stabilized with K strings. RESULT: The patient had dysesthesia which she experienced as cramps and persisting pain [Numeric Pain Rating Scale (NPRS) = 8/10 in the prior week] with acute episodes of 10/10 on the NPRS in the prior week. The patient was unable to stand, shift weight, or walk without persisting pain despite having an analgesic epidural catheter. Because of the pain, she was dependent on ADL. She was unable to complete her studies or sustain a romantic relationship. This greatly diminished her overall quality of life.
June 2012	On June 28, 2012, a below left knee amputation was performed. She was inpatient a rehabilitation department from July 11 to September 26, 2012. Due to the significant pain, the patient no longer ate and lost weight. During the hospitalization, she was unable to adhere to rehabilitation because of an altered level of consciousness due to the opioid treatment. Conventional rehabilitation was started with mirror therapy as soon as she was able. The conventional rehabilitation encompassed: - Care of the stump preparatory to prosthesis with surgical wound dressings - Scar removal massage - Stump bandaging - Strengthening of the pelvic girdle muscles - Skin desensitization of the stump and thigh - Global postural alignment - Joint mobilization of the knee and hip - Mirror therapy (2 months—not successful due to increased pain) - Re-education in standing posture and movement with a walker or crutches During hospitalization the patient showed significant phantom limb pain symptoms. She visited the pain therapy clinic, and they attempted modifications to medication with little benefit. Upon discharge, due to poor pain control and hyperalgesia of the stump, prosthesis of the left leg was not deemed feasible. RESULT: The mirror therapy was not successful. She had difficulty adopting this therapeutic approach because the motor imagery of her amputated limb was severely affected. She reported her ankle and toes felt blocked and not moving. She was unable to imagine her phantom limb moving when looking at her other leg through the mirror. Additionally, the effort to perform those exercises exacerbated the pain. The therapy was halted after 2 months, and a prosthetic device was not recommended because of persistent phantom limb pain and persistent allodynia in the stump. At discharge, the patient underwent a detoxification treatment for opioid addiction with methadone hydrochloride (10 ml, 2×/day), an antidepressant (duloxetine, 60 mg, 1 pill/day), and fentanyl (25 mcg/h every 60 h).
October 2012	Three months and two days after the amputation, the patient was admitted as an inpatient in the Centro Studi di Riabilitazione Neurocognitiva (Study Center of Cognitive Multisensory Rehabilitation), Villa Miari, Santorso, Italy for 7 weeks of “Cognitive Multisensory Rehabilitation”, twice a day, 5 days/week to treat PLP. The patient attended all sessions and tolerated CMR well. There were no adverse or unanticipated events.
December 2012	The patient's pain was reduced impressively, her sleep quality was improved, and she could don a simple homemade prosthesis made of foam rubber. She was able to do a few steps on the parallel bars while giving a partial load on this prosthesis. The stump could now be touched so she could wear this homemade prosthesis. The patient was discharged as an inpatient and the CMR therapist recommended she continue CMR sessions as an outpatient in her hometown with another CMR therapist for another 3 weeks. (Total CMR intervention time = 2.5 months). The patient learned to walk correctly with a normal prosthesis and two crutches.
May 2013	The patient could walk correctly with a normal prosthesis and a crutch.
August 2013	The patient could walk correctly at normal speed without crutches.
September 2013	She no longer took Methadone or Fentanyl.
Since the CMR outpatient therapy	After the CMR therapy ended, she graduated, got married, had a daughter and works as a physical therapist.
March 2024	She reports that the PLP is under control, sometimes it flares up, especially when the prosthesis is removed in the evening or at night and during ovulation. In that case, she finds benefit by doing CMR exercises on her own using multisensory motor imagery as she has learned during therapy. She no longer takes any pain medications and the epidural stimulator is off. She walks independently with the prosthesis without assistive devices. She is now pregnant with her second child.

### 2.1 Assessments and findings before the CMR intervention

PLP was measured with three pain scales. The IDentification Pain (ID Pain) questionnaire is a screening method to detect neuropathic pain, and is rated between −1 and 5 with higher scores more indicative of neuropathic pain (41). The Numeric Pain Rating Scale (NPRS) measures pain intensity, ranging from 0-no pain to 10-worst pain imaginable (42, 43). The minimal clinically important difference for the NPRS is 30%–36% reduction in pain, reflected by 1.7–1.8 points (42, 43). The McGill Pain Questionnaire is a reliable and valid patient-reported outcome to measure and describe qualitative aspects of pain (44, 45). The McGill Pain Questionnaire encompasses a sensory pain dimension (e.g., tingling); an affective dimension (e.g., tiring); an evaluative dimension (e.g., unbearable); and a miscellaneous dimension (e.g., nagging). We used the scoring system as described in Melzack (1975) for the Pain Rating Index based on the Rank values of the words [PRI (R)] and reported the total score for all categories, ranging from 0-no pain to 78-severe pain (45). The word in each subclass implying the least pain is given a value of 1, the next word is given a value of 2, etc (45). The patient chose those words that best described her pain experience.

At intake, the patient scored 5/5 on the ID Pain. When asked about pain in the prior week, she reported continuous pain with NPRS scores of 6.5/10 on average during the day and 8.5/10 at night, affecting her sleep quality. She had frequent episodes of 9.5/10 day and night. She scored 39/78 on the McGill Pain Questionnaire and described her pain as tremors (“everything is shaking”), pins and needles, stings, tingling, itching, blades piercing, pulsing, tremendous electric shocks, with pain worsening when the stump was touched. Since she could not control the pain, she tried to ignore the PLP and continued activities as much as possible. The pain levels and pain medication intake at admission are listed in Table 2.

**Table 2:** Evaluation of pain outcomes during CMR intervention.

Pain outcome	Admission 10/29/2012	Discharge 12/17/2012	Follow-up 09/15/2013	Follow-up 3/27/2024
NPRS (0–10)	Continuous PLP 6.5/10 average pain in the past week, i.e., pain experienced most of the time during the day, with 8.5/10 on average at night. Very frequent, very intense maximum pain of 9.5/10 during the week which felt like electroshocks.	No more PLP Just “muscular pain” ranging from 4 to 5.5/10 (average pain in the past week), occurring after exercises.	NA	PLP is under control. Sometimes PLP flares up, on average once week, especially when the prosthesis is removed in the evening or during the night (NPRS 4/10), and during ovulation (NPRS 7/10). When this happens, doing CMR exercises on her own using multisensory motor imagery, as she learned in the therapy, is effective in bringing down the pain within 10–15 min.
McGill Pain Questionnaire (0–78) PRI(R) total score	39	5	NA	13
ID pain (0 to 5)	5	0	NA	5 (when pain is present which is +/- 1×/week)
Epidural stimulator	on	off	off	off
Pregabalin (Lyrica)	300 mg, 2×/day	150 mg, 2×/day	150 mg, 2×/day (prescribed for another year)	None
Methadone	10 mg, 2×/day	3.5 mg, 2×/day	None	None
Fentalgon (Fentanyl)	25 mcg (1 patch) every 60 h	12.5 mcg (0.5 patch) every 60 h	None	None
Cymbalta	60 mg, 1×/day	None		None

Pain rating index based on the rank values of the words [PRI (R)] and reported on the total score for all categories: The word in each subclass implying the least pain is given a value of 1, the next word is given a value of 2, etc. For example, for the McGill Scoring on 10/29/2012, the patient scored 21 on the sensory subclass, 7 on the affective subclass, 4 on the evaluative subclass, and 7 on the miscellaneous subclass, for a total of 39 points.

Cognitive Multisensory Rehabilitation (CMR) provides multisensory discrimination exercises on the healthy side and multisensory motor imagery exercises of present and past actions on the amputated side to restore MBR and consequently reduce PLP. Multisensory motor imagery of (present or past) actions is defined as a unitary construct deriving from multiple information modalities: somesthetic (tactile, kinesthetic, pressure…), and visual information.

As part of the CMR evaluation, the therapist inquired about the patient's ability to imagine the feeling of meaningful non-painful actions done in the past (i.e., multisensory motor imagery), before pain onset. Despite numerous attempts and suggestions, the patient was unable to recall a memory that involved her left foot. She reported: “I cannot remember what I felt when I was trying on those white boots I wanted to buy”; “I cannot imagine doing anything with my left foot, not even feeling its contact with the floor. The ankle is locked.”; or “If I try to remember, for instance, what I felt when climbing the stairs in my house, I can feel only the right foot, the left foot is not there.” Attempting to imagine her left foot in the present moment increased her pain. On the other hand, she was able to perform multisensory motor imagery of the healthy foot but failed to correctly perform multisensory discrimination exercises with the healthy foot.

## 3 Diagnostic assessment: cognitive multisensory rehabilitation (CMR)

As inpatient, the patient received a total 35 CMR, 2×/day, 5 days/week for 7 weeks (10/29/2012–12/17/2012). The CMR exercises were organized in three specific steps, each with specific goals. First, exercises focused on multisensory motor imagery of the healthy limb based on the perception of the healthy limb during CMR exercises (construction of tactile, kinesthetic, and pressure information). Then, focus was on restoring somesthetic memories of the (now amputated) limb in a healthy state before the onset of pain that led to amputation. Once these memories were recalled correctly, the patient could use this information to improve the multisensory motor imagery of the missing limb in its current state, which is a gradual and complex process. For example, reflections from the patient early on were vague, demonstrating incomplete somesthetic imagery: “I feel like on a cloud, as if the foot is not resting on the floor”; or later, when exercises focused more on perception of contact of the sole of the foot with the floor, the patient referred to her perception as, “not exactly the same between the two feet. I feel the skin on the right sliding and modifying, while on the left the skin feels in one piece, smoother but stiffer, as if my foot didn't have its regular features”. These descriptions guided the CMR therapist to design specific exercises to help recover MBR. More details of the CMR exercises designed for this patient are presented below and in the Supplementary Material.

### 3.1 Step 1: Obtaining a correct perception of the multisensory information from the healthy right foot while interacting with the environment

Exercises 1–4, Figure 1–4B (in the Supplementary Material) were designed to restore the patient's ability to perceive tactile, space, pressure, weight, proprioceptive, and somesthetic information from the healthy (right) foot correctly and coherently. The reasoning for these exercises is that once the patient can focus and perceive the information from the healthy foot and ankle correctly and coherently with guidance from the therapist (with questions like “Have you ever felt anything like this in the past?”), the therapist will be able to focus on recovering memories of perceptions related to her left foot in actions performed before pain onset, and then on regaining multisensory imagery of her missing limb in the present moment. CMR therapists assume that it is important to be able to imagine the missing foot in the present moment as if it were present at that time in a healthy state.

### 3.2 Step 2: Retrieving memories of past performances

Once Step 1 was correctly performed, the patient was asked to find memories of past actions in which perceptions, experienced during exercises in Step 1, were felt in both feet. The therapist's questions are mentioned in Supplementary Material (Exercises 5–7, Figures 5–7). Examples of questions to help restore MBR were: “Have you ever had this feeling in the past in any action? With your left foot, as well?” The therapist then analyzed the way the patient described the images and identified potential flaws in those images caused by her condition. For example, at the start of CMR, the patient could only imagine the forefoot in an equinus foot position when in contact with the floor. She reported feelings of “strain” and of “being locked in the ankle” and described this experience as: “I remember I was sitting on a chair at University and tipping the chair while keeping the chair balanced by using the tip of the foot to block the chair from going overboard.” To help correct these MBR deficits, the therapist asked the patient to *compare* perceptions experienced during exercises in Step 1 with those of the image just retrieved of balancing the chair. Different parts of the foot are involved in these two examples (i.e., forefoot to balance the chair vs. whole foot contact during Step 1). By making the comparison between past and present experiences of motor imagery of the left foot, the patient realized that she could only imagine past actions with the left forefoot, not the whole foot.

In Exercise 5, Figure 5 (in the Supplementary Material), the therapist placed a weight on the board either in front of the toes or behind the heel, and asked the patient to keep the board horizontal and to perceive where the pressure was the greatest (forefoot or heel) and if there was a difference in pressure between the forefoot and the heel. This exercise was designed to improve the multisensory motor imagery that the patient had just described. The patient had to distinguish differences in pressure/weight under the sole of the healthy foot and proprioceptive and somesthetic information provided by the ankle by having her foot placed on a balance board.

In Exercise 6, Figure 6 (in the Supplementary Material), the patient compared two sponges placed under the right forefoot and one under the heel. The patient had to analyze the sensations of the parts of the foot that were in contact with sponges of different firmness to perceive its characteristics. At this point, the patient was able to perceive the soft sponge pushing in between her toes and the sponge that was wrapped around the heel. She reported that the skin of the sole of the right foot stretched when the heel felt pushed into the sponge.

### 3.3 Step 3: Comparing perceptions between the two limbs

The therapist then prompted the patient to *compare* this new insight to memories of actions where similar feelings were felt before pain onset, for example during walking. The patient reported that she remembered a feeling similar to the exercise when she was walking on the beach: her foot sank in the sand, and the sand slipped in between her toes. While remembering this, her foot felt flexible. This was the first time the patient was able to recall a normal feeling of her left foot, without any stiffness, fatigue, or pain.

The therapist asked “Can you imagine now the same feeling in your left foot as you are feeling in your right foot? Are the feelings exactly the same? Have you ever felt this kind of feeling in the past? Are the current sensations the same as in the past?” Comparing the left and right foot was a fundamental strategy at this time in the rehabilitative process. In Exercise 7, Figure 7 (in the Supplementary Material), the patient confirmed the remembered feeling in the left foot from walking in the sand was the same as in the right foot during the exercise. The patient also felt a comfortable warmth in the (imagined) left foot, which provoked an emotional reaction because she had not felt that in such a long time. By remembering her healthy left foot from the past, she could perceive her missing foot correctly in the present without PLP.

### 3.4 Progress during CMR as inpatient

With time, the patient improved in processing multisensory information in the healthy foot, and in multisensory motor imagery of the amputated foot regarding past actions and the present moment. Based on that progress, more targeted exercises were given to develop a complete mental representation of the internal parts of the left foot. Even though the patient showed progress, she still lacked perception of the toes. She reported: “I can feel the heel and under the middle of the foot (metatarsal heads), but I cannot feel my toes”.

One month after starting CMR, the PLP's intensity and frequency were reduced, and the stump could be touched without evoking pain. A homemade prosthesis was donned to introduce exercises in standing position and begin weight shifting on the amputated leg. The patient was discharged on 12/17/2012. She continued CMR in her hometown as an outpatient for another 3 weeks to achieve full recovery of the left foot representation. In total, the patient received 2.5 months of CMR.

### 3.5 Assessments and findings after CMR

At discharge from the Study Center of Neurocognitive Rehabilitation, the patient's quality of life was greatly improved. She only reported “muscular pain” of 4–5.5/10 after exercise on the NPRS scale. She did not experience neuropathic pain anymore. She scored 5/78 on the McGill Questionnaire and 0/5 on the ID Pain. The epidural stimulator was off. Medication was reduced to ⅓ Methadone, ½ Lyrica, and ½ Fentalgon (Fentanyl) dosages compared to baseline. Further gradual medication reduction was planned.

Table 2 shows the progression of pain measures and medication intake at admission, discharge, 10-month, and 12-year follow-up. The patient was able to control her pain by following the CMR exercise instructions. She slept through the night. In therapy around the time of discharge, she could perform CMR exercises in a standing position with the homemade prosthesis. At home, she donned a normal prosthesis without PLP, and learned to walk with the prosthesis and two crutches. In May 2013, she could correctly walk with the prosthesis and one crutch. In August 2013, she could walk correctly, at normal speed, with the prosthesis and without crutches. In September 2013, she walked normally and independently indoors and outdoors with her prosthesis without assistive devices, and she no longer took Methadone or Fentanyl. After the CMR therapy ended, she graduated, got married, had a daughter, and now works as a physical therapist. At present (March 2024), she reports the PLP is under control. Sometimes PLP flares up, on average once week, especially when the prosthesis is removed in the evening or during the night (NPRS 4/10), and during ovulation (NPRS 7/10). When this happens, doing CMR exercises on her own using multisensory motor imagery, as she learned in the therapy, effectively brings the pain down within 10–15 min. She no longer takes pain medications, and the epidural stimulator is off. She walks independently with the prosthesis without assistive devices and is pregnant with her second child.

## 4 Discussion

This case study reports on a patient with debilitating PLP and an inability to recall activities involving the missing limb. Even though altered motor imagery after amputation has been reported (46), to our knowledge, this is the first time that the inability to imagine actions with the limb before amputation has been reported. Once visual and multisensory imagery of past and present motor actions in both limbs were restored, the pain was alleviated, and the patient was able to don a prosthesis and walk.

While inability to imagine past actions with a (now) amputated limb seems to be understudied, we do not consider this to be a unique or rare phenomenon as we have seen that adults with SCI also have difficulty remembering past actions or even have difficulty visualizing (visual imagery) or feeling the parts of the body (kinesthetic imagery) that have sensory loss while imagining actions in the present moment. The latter was observed when testing patients with the Kinesthetic and Visual Imagery Questionnaire (KVIQ) (47).

The present work shows the feasibility of how CMR can open up new perspectives of treating PLP. The uniqueness of this approach is that the therapist provides cues to patients through questions whereby the patient is invited to make comparisons between past and present actions, between the multisensory imagery or tactile sensations in the remaining limb and imagery of the amputated limb, so they can recover visual and multisensory motor imageries of the body, on both the healthy and amputated side (48). Comparisons between both sides are made in terms of perceptions of multisensory information in the present moment, imagery of actions in the present and the past, as well as comparing past actions with present actions (48).

While we did not perform brain imaging in this case study, our knowledge from prior research (29, 30), as well as this patient's feedback describing her body, leads us to assume she was able to recover her MBR. More specifically, she was able to produce a current multisensory motor imagery of her body along with the missing limb and produce correct memories and multisensory imagery of actions performed with the limb before the amputation. Based on our prior research, we hypothesize that these improvements are due to the restoration of the multisensory integration and MBR, possibly reflected by the reorganization of sensorimotor cortical areas, thereby reducing PLP (27, 31, 49).

When comparing our CMR research to reduce PLP to our CMR research to reduce neuropathic pain in adults with SCI, we hypothesize that a similar mechanism of recovery is at work. We acknowledge that adults with SCI still have a visual perception of the limbs. However, that is only if they look at the limbs. If they are sitting at a table, with their legs under the table, their brain may be as confused as those with amputated legs because some participants do not have feeling anymore in those legs. For example, in our prior studies in adults with SCI, some participants stated, “It is as if I am living from the waist up”.

We encourage further research in a larger sample to validate the findings that restoring (i) memory and multisensory imagery of past actions when both limbs were healthy, (ii) restoring visual and somesthetic motor imagery in both limbs in the present situation, and (iii) comparing the somesthetic motor imagery of one limb with the other, can reduce PLP in adults with amputations.

## Data Availability

The original contributions presented in the study are included in the article/**Supplementary Material**, further inquiries can be directed to the corresponding author.
