# Supplementary material for: Case Report: Phantom limb pain relief after cognitive multisensory rehabilitation
Source: Front Pain Res (Lausanne). 2024 Apr 25;5:1374141. doi: 10.3389/fpain.2024.1374141 (PMC11079144; doi:10.3389/fpain.2024.1374141)
Supplement: Supplementary file 1 [file Datasheet1.docx]

Supplementary Material

**Supplementary Table. Examples of CMR exercises (STEP 1-3)**

| **STEP 1: Obtaining a correct perception of the tactile, space, pressure, weight, proprioceptive, and somesthetic information from the healthy right foot while interacting with the environment.** | |  |
| --- | --- | --- |
| Exercise 1 | The therapist moves the healthy foot forward and backward on a wooden surface and asks the participant to recognize with eyes closed the spatial relationship between the knee and the foot. The therapist asks, “Is the knee over your ankle, over the middle of the foot, or the toes?”  To successfully perform the task, the participant needs to attend to the somesthetic information from the knee and ankle, and to the changing pressure under the sole of the foot.  Questions/Suggestions provided by the therapist to help the patient restore her mental body representations: “Can you feel how the ankle has changed? Pay attention to how the weight distribution under your foot changes. Is the weight of your leg distributed more under the heel, under the ball of the foot, or is it distributed evenly under the entire foot?”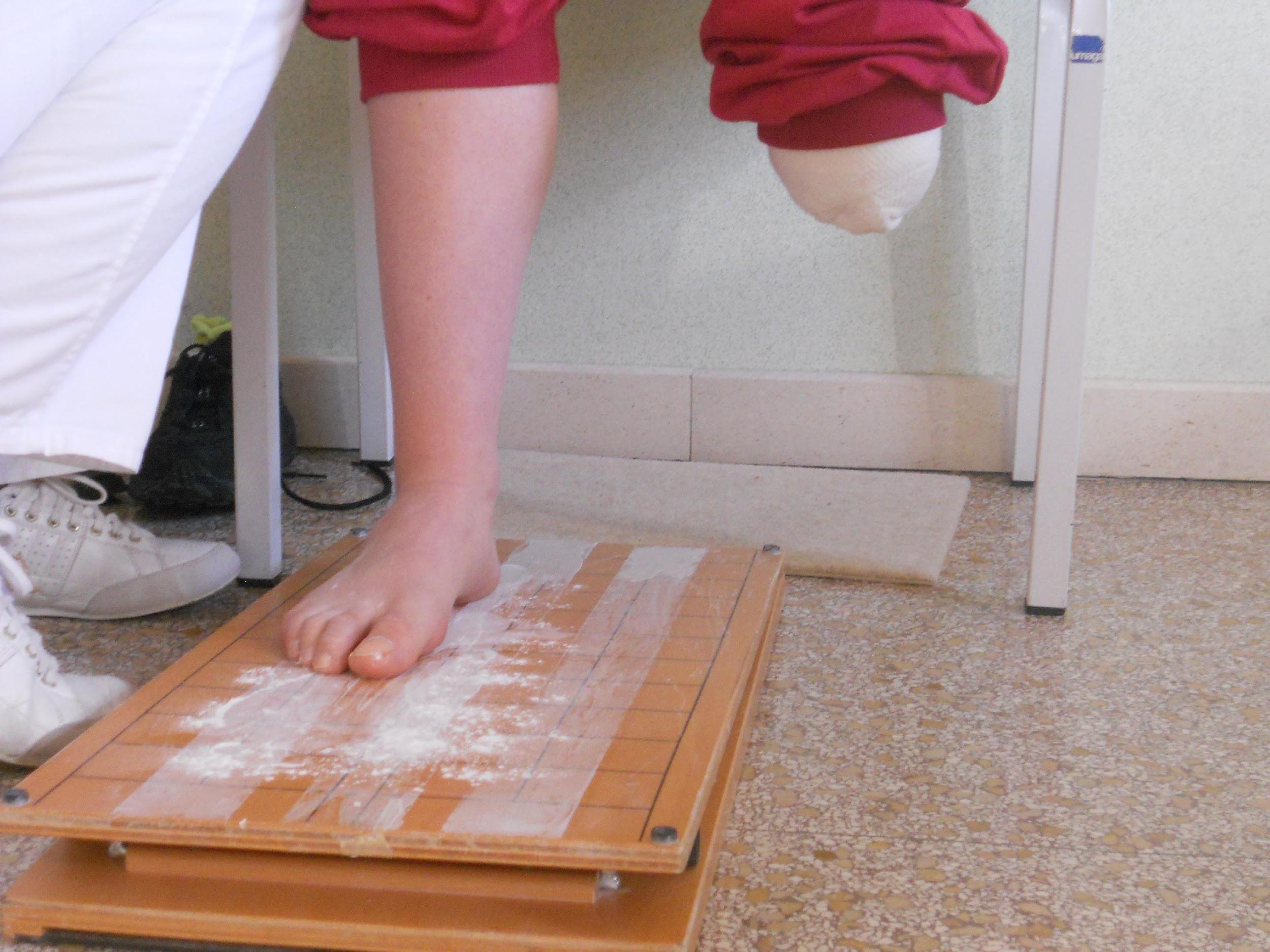  **Figure 1.** Exercise of recognition of the spatial relationship between the knee and the foot on the healthy side. |  |
| Exercise 2 | The therapist places one or more textures under the healthy foot and asks the participant to recognize them with eyes closed. The therapist asks:  “Is there only one type of surface under your foot or more? Which one/ones? Can you describe it/them?”  To successfully perform the task, the participant needs to attend to the tactile information under the sole of the foot.  Questions/Suggestions provided by the therapist to help the patient restore her mental body representations: “Pay attention to the sole of your foot. Is the surface you feel under the sole of your foot homogeneous? Do you feel differences between the heel and forefoot? Pay attention to how the surface welcomes your foot: is it flat, smooth, hairy...?”  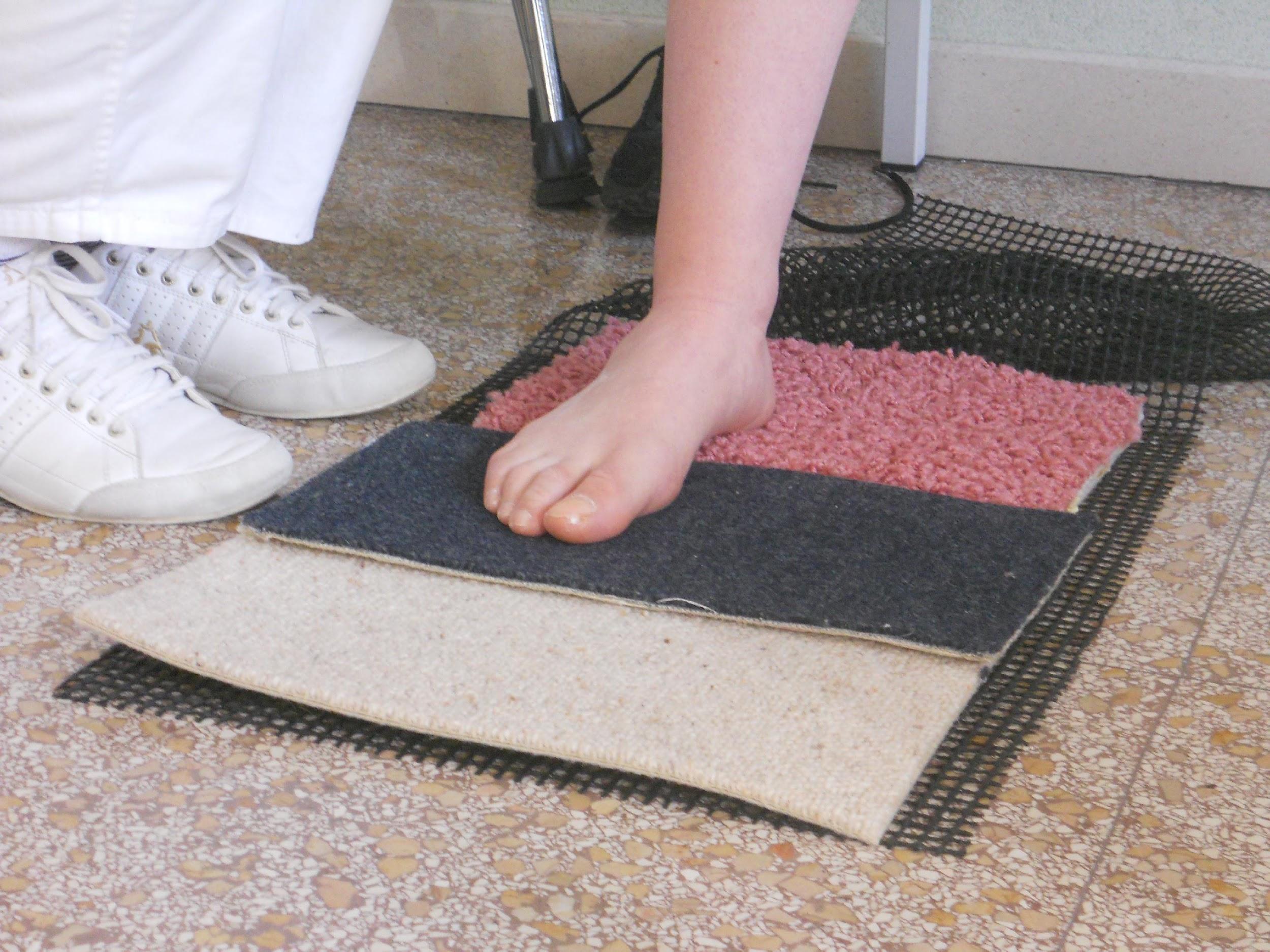  **Figure 2.** Exercise of tactile recognition under the sole of the healthy foot. |  |
| Exercise 3 | While performing a flexion-extension movement of the participant’s healthy ankle, the therapist places five sponges of different firmness under her heel, one after the other in random order, and asks the participant to recognize them with eyes closed. The therapist asks: “Which one of the 5 sponges are you perceiving?”  To successfully perform the task, the participant needs to attend to and integrate the pressure information under the heel and somesthetic information from the ankle.  Questions/Suggestions provided by the therapist to help the patient restore her mental body representations: “To understand which sponge it is, pay attention to how much sensation the ankle feels and how consistent the feeling is around the heel: Is it a soft sponge that welcomes you and envelops you, or is it a more rigid sponge that supports you?”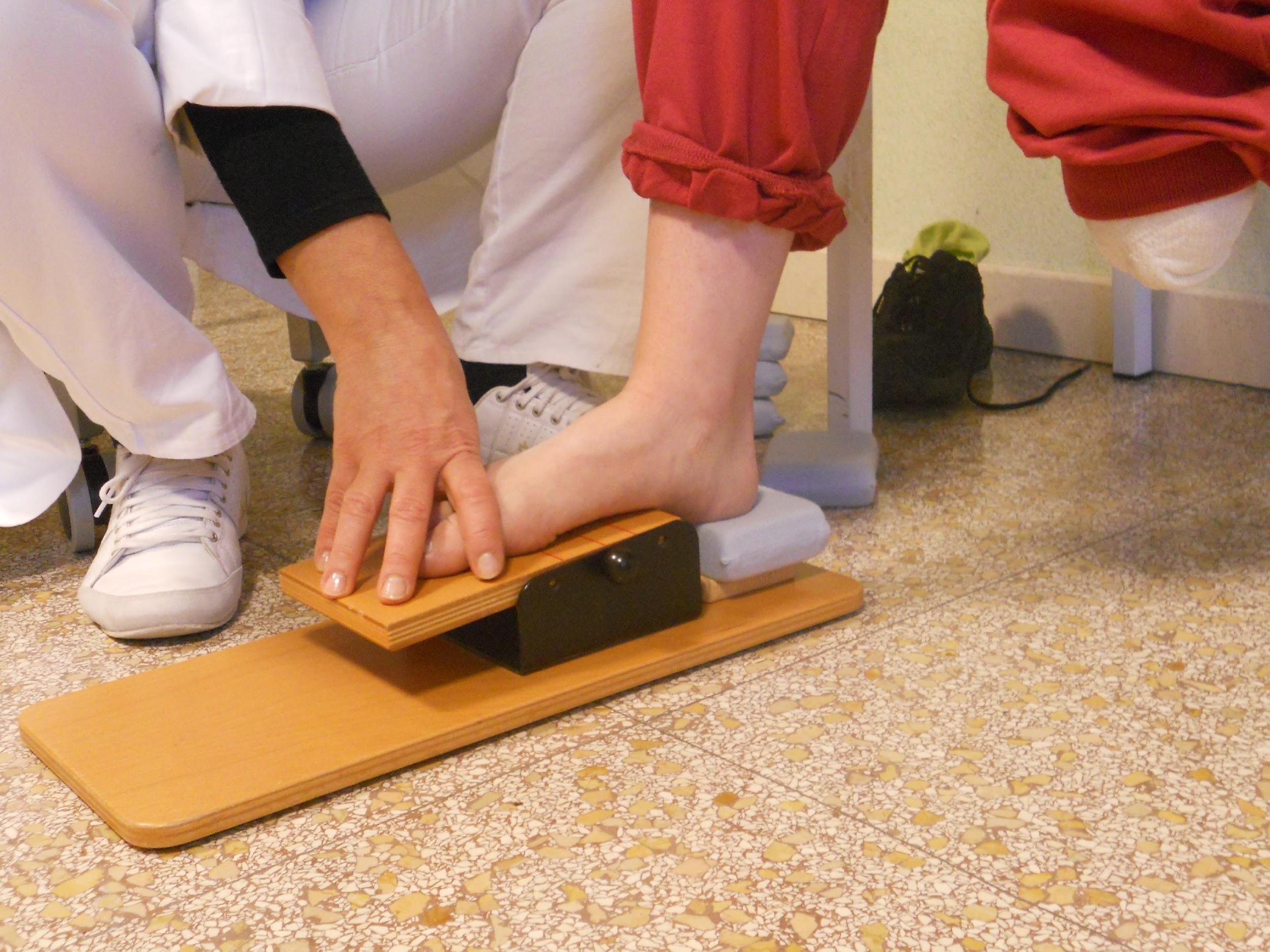  **Figure 3.** Exercise of recognition of firmness of sponges/pressure under the heel of the healthy foot. |  |
| Exercise 4 | The therapist moves the participant’s healthy leg and foot into various positions, pivoting on the hip, knee, and ankle, while keeping the foot above the ground. The therapist then asks: “Can you tell me which part of your foot will touch the floor first? Where will you feel more pressure when the foot is in full contact with the floor?”  To successfully perform the task, the participant needs to anticipate the feelings of touch and pressure in the foot, while paying attention to somesthetic information from the hip, knee, and ankle.  Questions/Suggestions provided by the therapist to help the patient restore her mental body representations: “Pay attention to how your hip, knee, and ankle change to bring your foot to touch the floor. Which part of the foot do you think will touch the floor first? Now imagine your foot in full support. How will the weight be distributed under the sole of your foot: Uniform, or more concentrated under one part of the foot (heel, forefoot, internal or external portion of the foot or part of it)?”  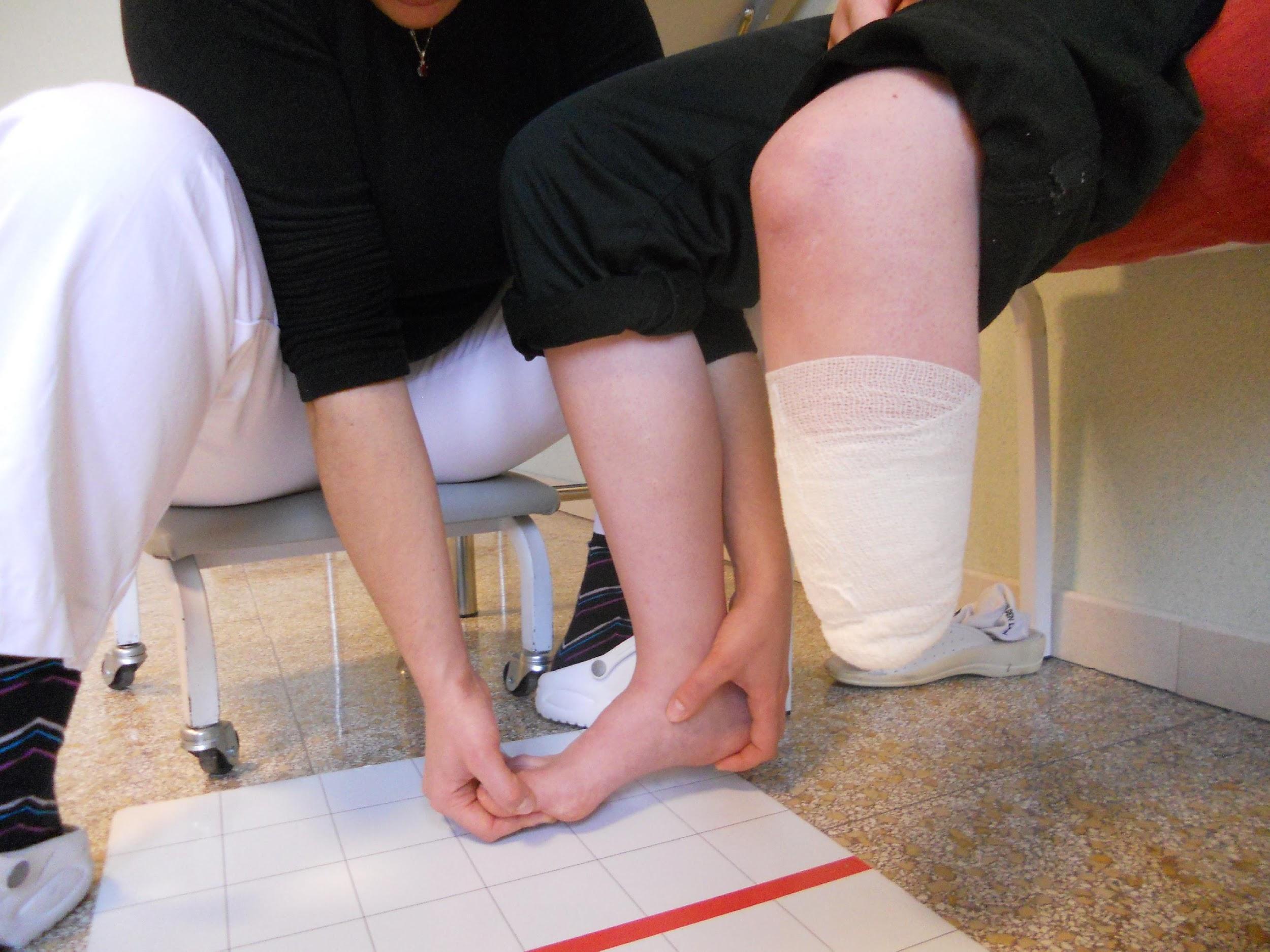 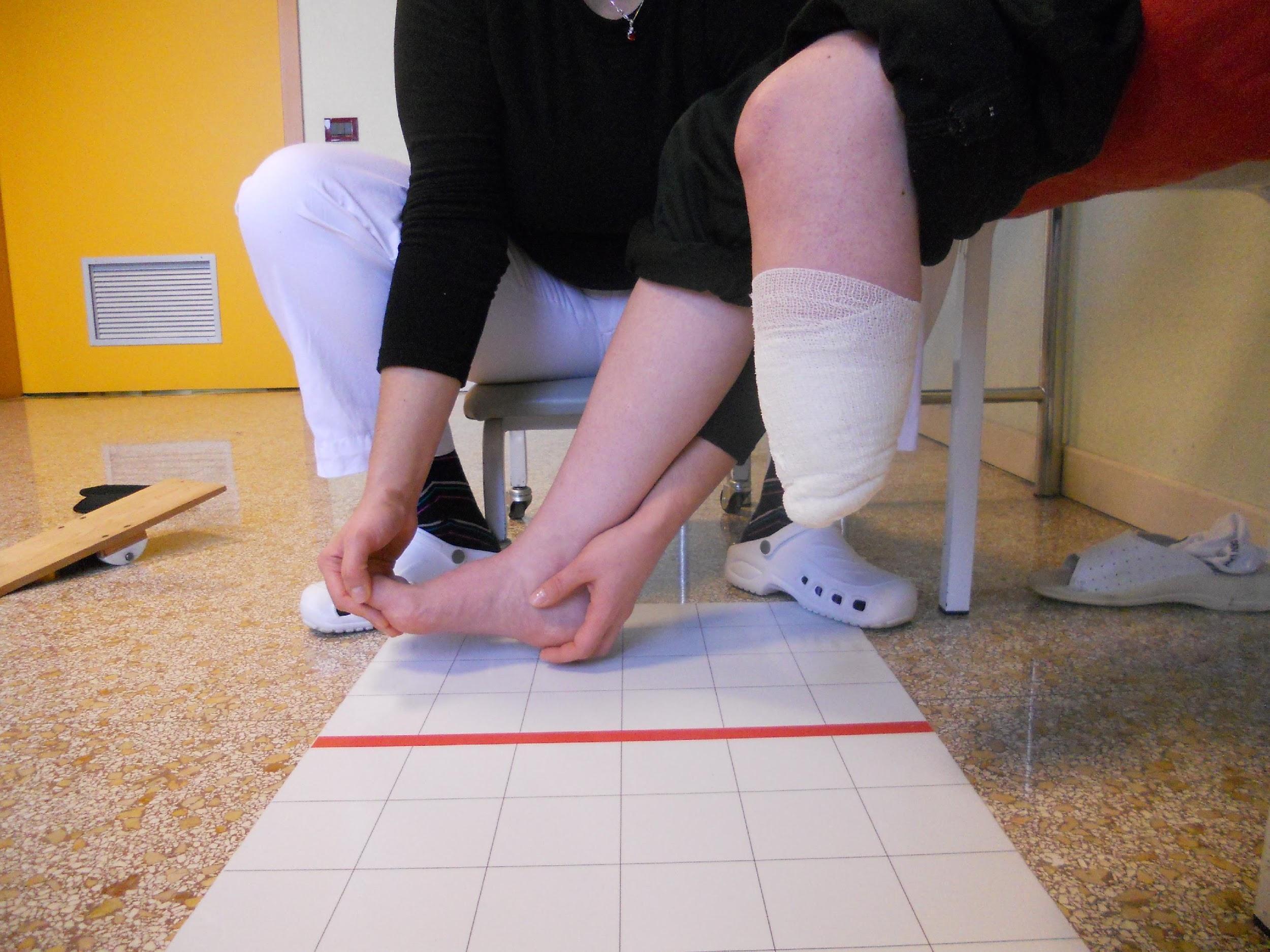  **Figures 4A and 4B.** Exercises of anticipation of the contact of the foot to the ground through retrieving and integrating the proprioceptive and somesthetic information from the hip, knee, and ankle. | |
| **STEP 2: Retrieving memories of past performances.** | |  |
| Exercise 5 | The healthy foot is placed on a balance board.  The therapist placed a weight on the board either in front of the toes or behind the heel, and asked the patient to keep the board horizontal and to perceive where the pressure was the greatest (forefoot or heel) and if there was a difference in pressure between the forefoot and the heel. The questions were “Where can you feel more pressure, under your forefoot or under your heel? Is the difference in pressure big or small?”  To successfully perform the task, the patient needs to pay attention to and integrate the pressure and weight information under the sole of the foot as well as the proprioceptive and somesthetic information provided by the ankle.  Questions/Suggestions provided by the therapist to help the patient restore her mental body representations: “Pay attention to how the pressure under the sole of your foot changes. Where did it increase? Under the heel or under the forefoot? Do you remember ever feeling this sensation before your health issues?” 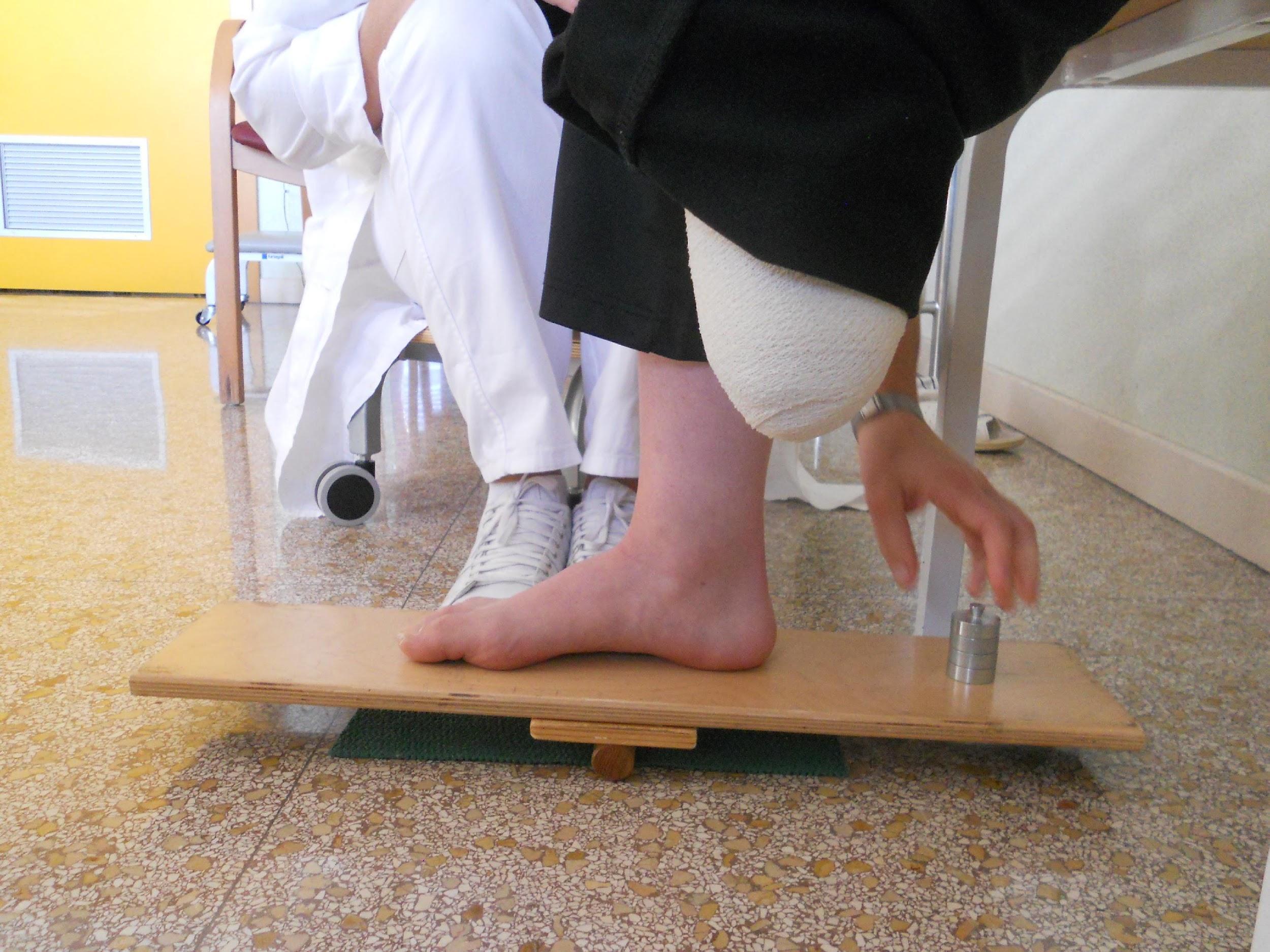  **Figure 5.** Exercise comparing pressure under the forefoot with pressure under the heel of the healthy foot on a balance board. |  |
| Exercise 6 | The therapist places three sponges, one under the heel, and two under the healthy forefoot. They may be the same sponges or different ones in terms of resistance felt. The patient is in a sitting position and has the eyes closed. The therapist asks: “Are they the same sponges or different? Can you tell me which type they are?”  To successfully perform the task, the participant needs to attend to and integrate the tactile and pressure information under the sole of the foot and the proprioceptive and somesthetic information from the ankle.  Questions/Suggestions provided by the therapist to help the patient restore her mental body representations: “Pay attention to your foot. Is the consistency of the three sponges homogeneous? Feel how the sponges accommodate or support your foot in every part of it. Try to feel if the inclination of the foot changes because of the sinking of one sponge. Have you ever felt this sensation in the past, before your health issues?”  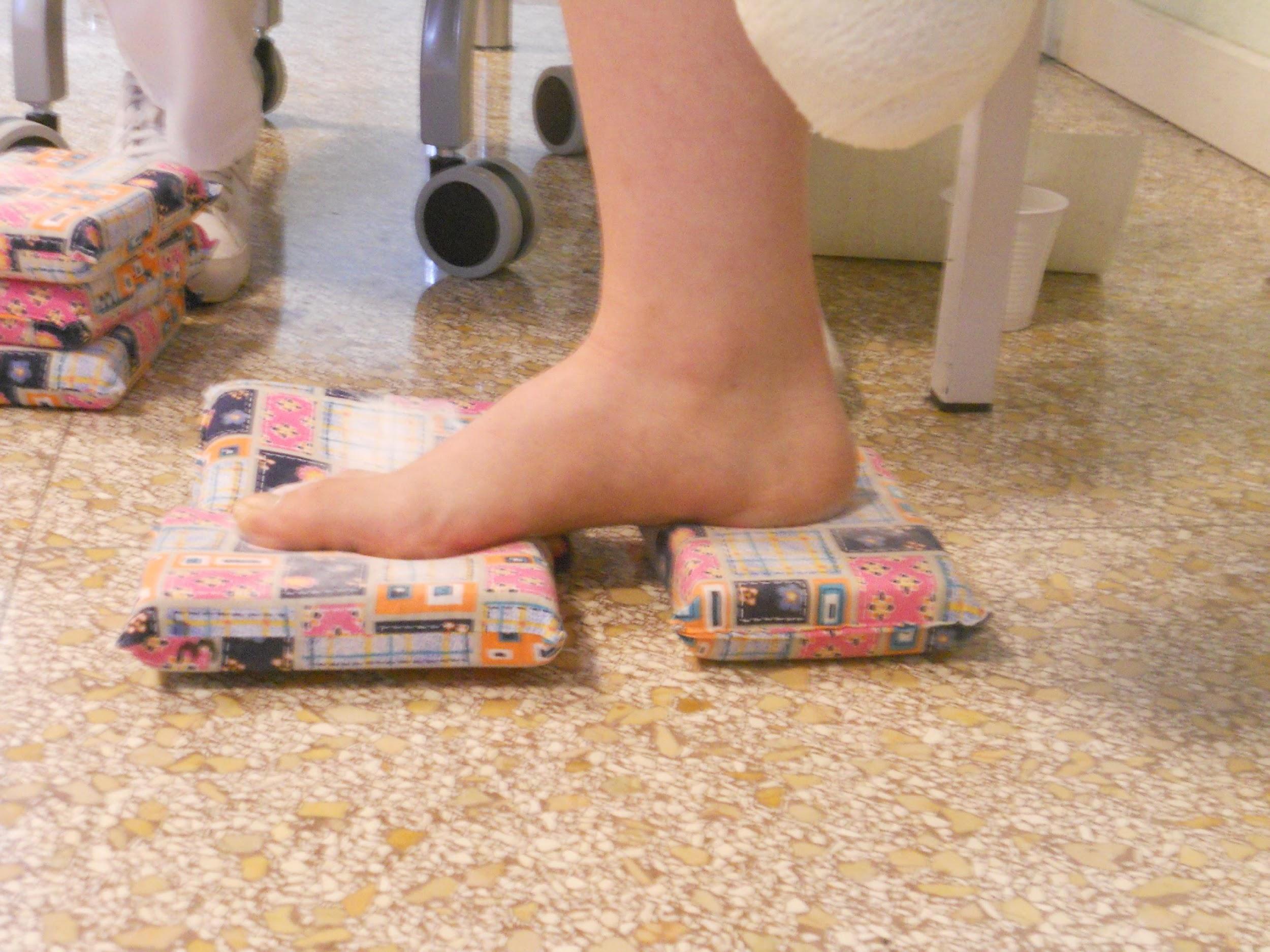  **Figure 6.** Exercise comparing pressure under the forefoot with pressure under the heel of the healthy foot, using sponges of different firmness. |  |
| **STEP 3: Comparing perceptions between the two limbs.**  For this step, all the exercises described above, or similar ones, can be used. For example: | |  |
| Exercise 7 | The healthy forefoot is placed on two sponges (one on the right and one on the left of the forefoot).  The question is “Pay attention to the two sponges you can feel under your forefoot and toes. Are they equal? If not, which of these is the softer?”  To successfully perform the task, the patient needs to pay attention to pressure information under the forefoot of the healthy foot.  Questions/Suggestions provided by the therapist to help the patient restore her mental body representations: “Pay attention to your forefoot. Is the consistency of the two sponges homogeneous? Feel how the sponges accommodate or support your forefoot. Do they do it the same way?”  **Then**, when the patient improves in distinguishing the different levels of firmness with the healthy forefoot and can answer the questions correctly, the therapist asks her to imagine doing the same exercise with the missing foot as well, imagining the feeling at the same time (multisensory motor imagery; recognizing the same movement, the same perceptions under the forefoot): “Can you imagine doing the same exercise at the same time with the left foot as well? Can you feel the same feelings in both your feet at the same time?”  Questions/Suggestions provided by the therapist to help the patient restore her mental body representations: “Can you imagine what you are feeling beneath your left foot in the same way as you are feeling beneath your right foot? Can you feel the same feelings in both your feet? Is the feeling the same?”  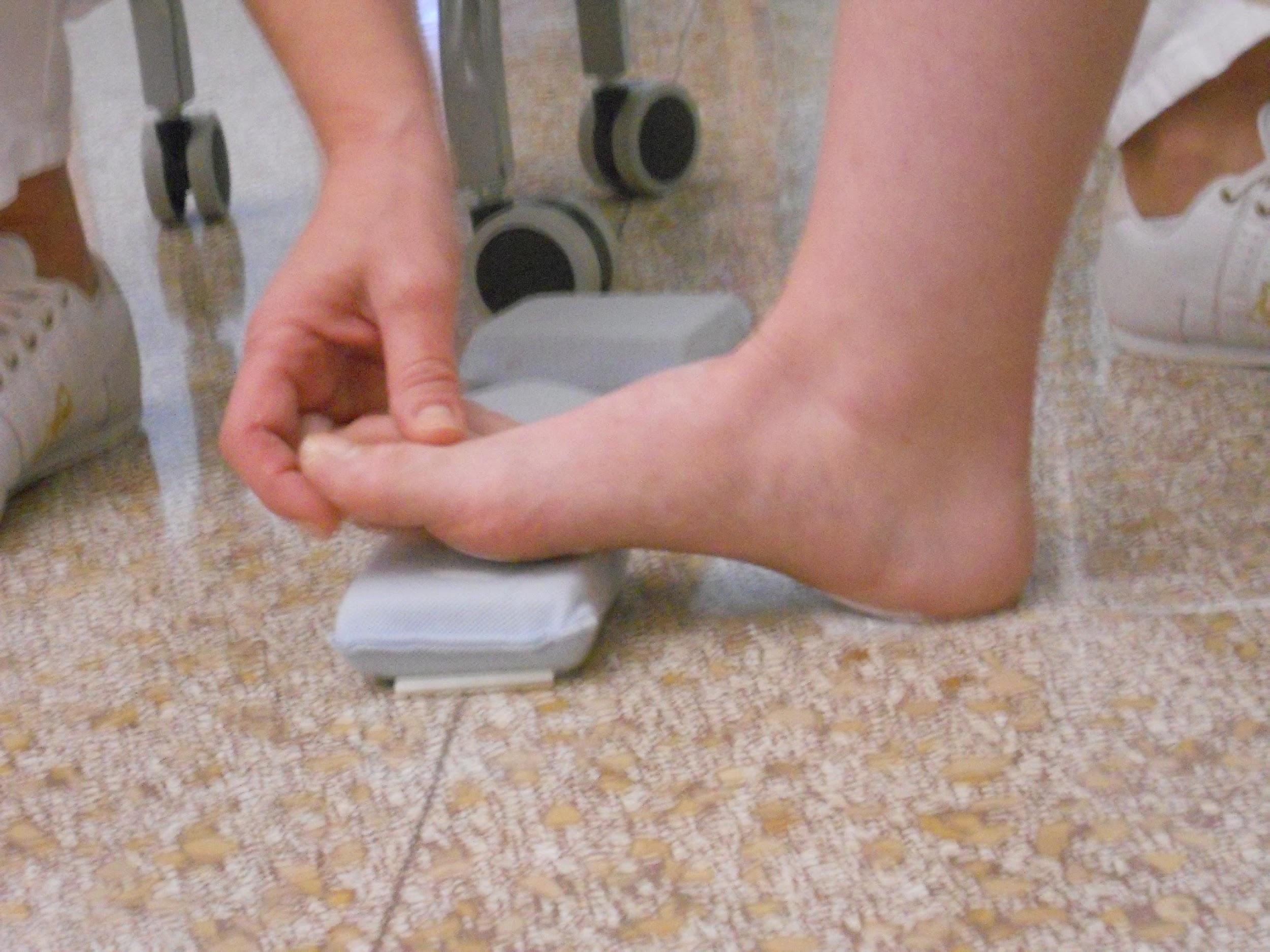  **Figure 7.** Exercise comparing the firmness of two sponges under the forefoot and comparing the sensation experienced under the right forefoot with the imagined (amputated) left forefoot. |  |
